# Antiphospholipid syndrome in the paediatric population: performance of ACR-EULAR classification criteria in a controlled cohort

**DOI:** 10.1136/lupus-2026-002013

**Published:** 2026-06-29

**Authors:** Stanley Niznik, Daniel Bar, Shiri Spielman, Rotem Semo-Oz, Assaf Barg, Sarina Levy-Mendelovich, Gili Kenet, Nancy Agmon-Levin, Irit Tirosh

**Affiliations:** 1 Zabludowicz Center for Autoimmune Diseases, Sheba Medical Center, Tel Hashomer, Tel Aviv, Israel; 2 Tel Aviv University Sackler Faculty of Medicine, Tel Aviv, Israel; 3 Pediatric Ward B, The Edmond and Lily Safra Children’s Hospital, Tel HaShomer, Israel; 4 Gray Faculty of Medicine, Tel Aviv University, Tel Aviv-Yafo, Israel; 5 Pediatric Rheumatology, The Edmond and Lily Safra Children’s Hospital, Tel HaShomer, Israel; 6 The Israeli National Hemophilia Center and Thrombosis Unit, Sheba Medical Center National Hemophilia Center and Institute of Thrombosis & Hemostasis, Tel HaShomer, Israel; 7 Clinical Immunology, Allergy And Urticaria, Sheba Medical Center, Tel HaShomer, Israel

**Keywords:** Antiphospholipid Syndrome, Antibodies, Antiphospholipid, Hematology

## Abstract

**Introduction:**

Antiphospholipid syndrome (APS) is a systemic autoimmune disorder marked by thrombotic and non-thrombotic manifestations. In children, diagnosis remains challenging due to lack of validated paediatric criteria. As the 2006 revised Sapporo has been used without validation, and the 2023 American College of Rheumatology/European Alliance of Associations for Rheumatology (ACR-EULAR) classification system based on a more modern disease improves specificity; however, their performance and applicability in the paediatric population have not yet been extensively vetted.

**Objective:**

This study evaluates the performance of the ACR-EULAR APS criteria in a paediatric cohort of APS and antiphospholipid antibody (aPL) patients, focusing on key diagnostic parameters and comparison with traditional criteria.

**Methods:**

A retrospective review of 69 patients’ ≤18 years followed at the Sheba Medical Center (2011–2024) was conducted. Clinical, laboratory and serological data including anti-cardiolipin, anti-β2GPI, lupus anticoagulant as well as APS score (adjusted Global Antiphospholipid Syndrome Score, aGAPSS) were analysed. Patients were divided into APS patients (n=29) or aPL carriers (controls; n=40). APS diagnosis was made based on expert opinion and validated by two independent experts.

**Results:**

In our cohort (mean age 16 years; 62% female). APS diagnosis was more common among those with a family history of APS (37.5% vs 17.5%; p=0.056). Comparing APS patients and aPL carriers, unprovoked venous thrombosis (OR 1.55, 95% CI 1.02 to 2.36) and aGAPSS OR 1.053 (1.031 to 1.076) and platelet number (OR 0.999, 95% CI 0.998 to 1) were the strongest predictors of APS. Moreover, non-thrombotic manifestations such as heart valve disease, livedo reticularis and diffuse alveolar haemorrhage were only present in the APS group. Catastrophic APS rate was 10.3% among our APS group. Mortality rate was 6.7% among APS group and 0% among aPL carriers.

In the paediatric population, the 2023 ACR/EULAR criteria demonstrated superior diagnostic performance compared with the 2006 Sapporo criteria, characterised by a higher positive likelihood ratio (18.23 vs 16.43) and a lower negative likelihood ratio (0.036 vs 0.136), effectively improving both the rule-in and rule-out capacity for APS diagnosis.

**Conclusions:**

The superior sensitivity and likelihood ratio of the 2023 ACR-EULAR criteria suggest they may be a valuable tool for the classification of paediatric APS; however, further prospective validation is essential to optimise their clinical application.

**WHAT IS ALREADY KNOWN ON THIS TOPIC**

- Limited data regarding classification criteria in the paediatric population, specifically in the era of newer American College of Rheumatology/European Alliance of Associations for Rheumatology (ACR-EULAR) antiphospholipid syndrome (APS) classification criteria.

**WHAT THIS STUDY ADDS**

- One of the first publications to present a matched controlled cohort of APS patients in children, comparing two populations who are positive for antiphospholipid antibodies, one group who have had a clinical event consistent with APS and the other did not.

**HOW THIS STUDY MIGHT AFFECT RESEARCH, PRACTICE OR POLICY**

- Might strengthen clinicians to use 2023 ACR-EULAR criteria in children, raise awareness of the severity of APS among the paediatric population.

## Introduction

 Antiphospholipid syndrome (APS) is a systemic autoimmune disease characterised by arterial, venous or microvascular thromboembolic events, obstetric morbidity and a wide range of non-thrombotic clinical manifestations.^1 2^ APS may present as a primary or secondary disease, mostly secondary to systemic lupus erythematosus (SLE).^3^ Data on the epidemiology of APS, particularly in the paediatric population, are limited. Epidemiological studies from the USA and Italy have reported prevalence rates of APS in the general population ranging from 17 to 50 cases per 100 000 individuals.^4 5^ A review from Italy showed an estimated prevalence of 2.5/100 000 children and an annual incidence of 0.2/100 000 children.^6^

The diagnosis of APS remains complex even in adult populations, as clinicians must rely on classification criteria in the absence of universally validated diagnostic standards. This challenge is further magnified in paediatrics; currently, the majority of published paediatric cohorts are defined based on the 2006 revised Sapporo criteria, despite these criteria being designed with adult APS patients in mind.^7^ These criteria were assumed to be applicable for the paediatric population though never validated.^8^ In 2023, the American College of Rheumatology (ACR) and European League Against Rheumatology published new classification criteria—ACR-European Alliance of Associations for Rheumatology (EULAR) criteria, which incorporated non-thrombotic manifestations such as thrombocytopenia, heart valve disease and microvascular involvement, livedo reticularis, nephropathy, diffuse alveolar haemorrhage, myocardial infarction with non-occlusive disease—MINOCA and adrenal haemorrhage. Moreover, obstetric criteria were made more stringent to improve specificity.^9^

### Objective

Considering the recently published ACR-EULAR classification criteria for APS, we aimed to evaluate their performance in a controlled cohort of paediatric patients, as well as to identify the key parameters in the diagnosis of APS in this population.

## Methods

In this retrospective study, data were collected from the Rheumatology, Immunology and Haematology clinic visits between the years 2011 and 2024 at the academic tertiary hospital, Sheba Medical Center.

Inclusion criteria were age at presentation <18 years and the persistent presence of at least one positive antiphospholipid antibody (aPL).

We accumulated epidemiological, clinical and laboratory data for all patients included in our study. Additionally, our cohort was divided into two groups based on the treating clinician’s decision: patients with APS syndrome (study group, validated by two additional experts) versus aPL carriers (controls), namely patients with at least one positive persistent aPL test but no supportive parameters for the diagnosis of the syndrome according to their treating physician. All patients in both the disease and control groups were managed at a tertiary referral centre and underwent systematic clinical evaluation. This included serial echocardiography and active monitoring for both thrombotic and non-thrombotic manifestations (eg, livedo, heart valve disease, haematologic abnormalities) as part of standard-of-care clinical coordination.

### Serology

The presence of anti-cardiolipin (aCL) and anti-β_2_‐glycoprotein I (aβ_2_GPI) of the IgG and IgM isotypes was measured by ELISA or by a multiplex system. The kits that were used were all commercial (ELISA—aB2GPI by AESKU Diagnostics (Wendelsheim, Germany) and aCL by Varelisa (Pharmacia Diagnostics, Stockholm, Sweden); Bioplex both aB2GPI and aCL by Bio-Rad, Hercules, California, USA). To satisfy the 2006 revised Sapporo criteria, B2GPI and aCL positivity were defined as antibody levels exceeding the 99th percentile (GPL or MPL units) according to the manufacturer’s reference range. In contrast, for the 2023 ACR/EULAR criteria, a threshold of >40 units was applied to define positivity.

Lupus anticoagulant (LA) activity was detected by coagulation assays in routine use at each centre and was consistent with the International Society of Thrombosis and Hemostasis guideline.^10^ The LA assays were modified in 2016; up until 2016, LA activity was measured by LA-responsive activated partial thromboplastin time aPL (by Stago, confirmed using the Actem FS Kit by Siemens, Erlangen, Germany), and from 2016, LA activity was measured by a combination of silicon clotting time and the use of the Russell Viper Venom Kit (by IL, Bedford, Massachusetts, USA). In case of anticoagulation treatment or spontaneous INR >1.5, patients’ plasma was mixed with normal plasma to reduce false positivity. Positivity was defined as single, double or triple positive according to the number of different positive tests obtained.^11^

### Adjusted global antiphospholipid syndrome score

In this study, we used the validated adjusted Global Antiphospholipid Syndrome Score (aGAPSS), which allots 3 points for dyslipidaemia, 1 for arterial hypertension, 5 for aCL antibodies IgG/IgM, 4 for anti-β2 glycoprotein IgG/IgM and 4 for LA.^12^ We have also calculated the proposed aGAPSS for Cardiovascular Disease (aGAPSS-CVD), which incorporates factors such as smoking, diabetes and obesity. This score aims to emphasise the risk of CVD in patients with APS.^12^

### Statistical analysis

The data were analysed using the jamovi programme, developed by The Jamovi Project (2025), specifically jamovi V.2.6 (Computer Software). This can be accessed at https://www.jamovi.org. For analysing between-group differences in discrete variables, either Pearson’s χ^2^ test or Fisher’s exact test (two-tailed) was employed.

Multivariate analysis was performed using the GAMLj module in jamovi (V.2.3). We used a generalised linear model framework, which is a robust extension of traditional regression that better accommodates clinical datasets with binary outcomes and complex interactions. This method was specifically chosen for its superior ability to maintain mathematical stability and provide precise ORs in specialised clinical cohorts with a focused sample size. Significance was set at p<0.05.

## Results

**Figure 1:**
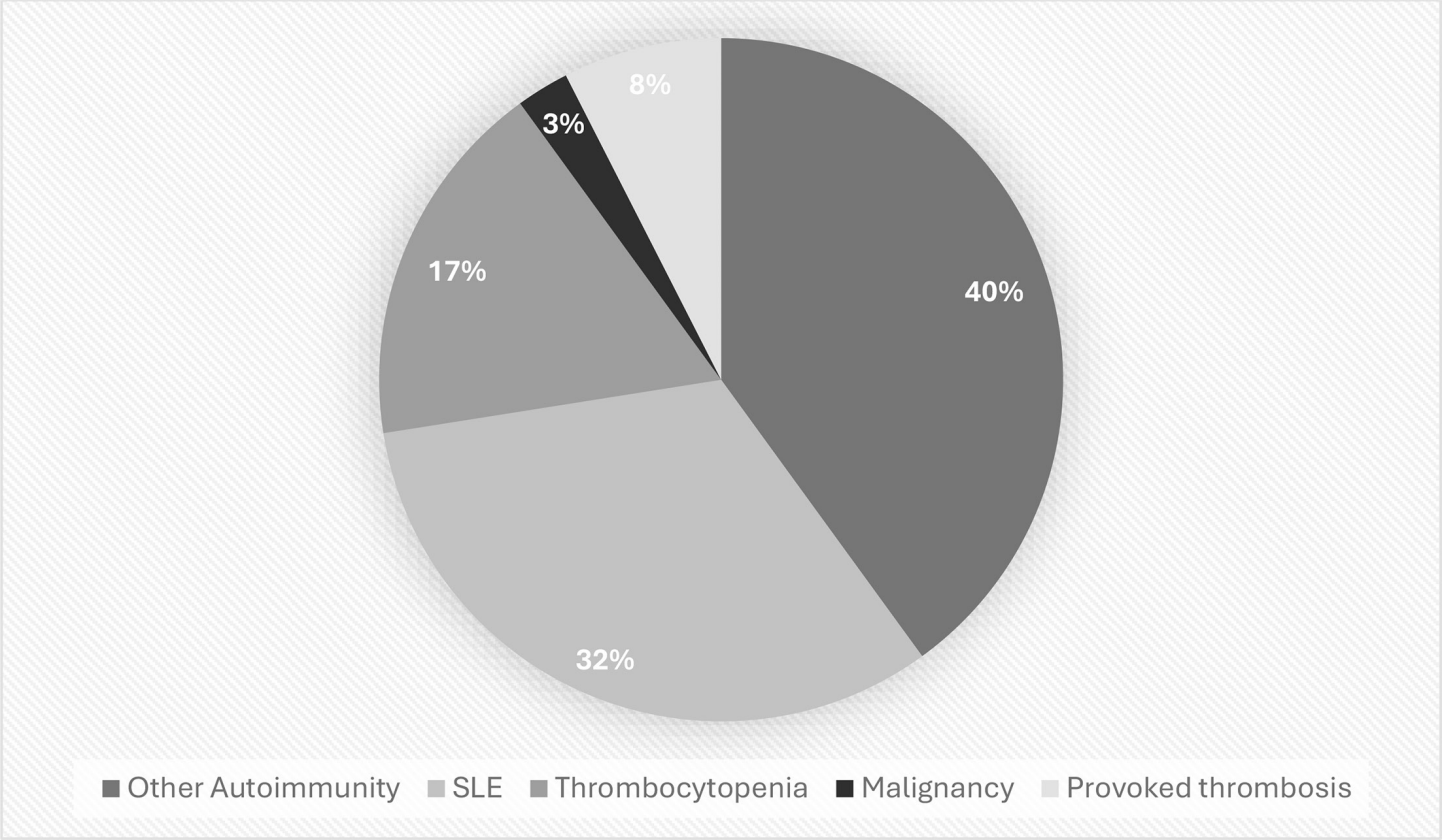
Division by diagnosis of control group (i.e. non APS patients). APS, antiphospholipid syndrome; SLE, systemic lupus erythematosus.

Our cohort consisted of 69 patients, 29 diagnosed with APS while 40 were considered as aPL carriers. Clinical scenarios that brought physicians to evaluate aPL antibodies were mainly other autoimmune conditions (ie, arthralgia with autoantibodies, autoimmune hepatitis, Sjögren, multiple sclerosis with CNS lesions), SLE diagnosis, thrombocytopenia, provoked thrombosis and malignancy (figure 1).

Among our APS group (table 1), the mean age at presentation was 16±2.1 with an average follow-up of 15.7±8.9, female proportion is 62.1% (female to male ratio 1.64). In our cohort, 37.9% of APS patients had a first-degree relative with an APS diagnosis, which is more than double the rate in the control group (17.5%, p=0.056). Notably, 58.6% of APS patients were diagnosed with primary diseases and 69% suffered from venous thromboembolic disease, while 34.4% suffered from arterial thrombosis (table 2).

**Table 1:** General Cohort parameters - APS Vs Controls

Parameter	APS (n=29)	Control (n=40)	P value
Age presentation	16 (±2.1)	13.9 (±3.7)	0.01
Follow-up	15.7 (±8.9)	10.2 (±6.1)	0.004
Sex (female proportion)	18 (62.1%)	29 (72.5%)	0.685
Primary APS	17 (58.6%)	NR	NA
Associated SLE	10 (34.5%)	20 (50%)	0.888
Other autoimmunity	4 (13.8%)	9 (22.5%)	0.404
First-degree relative with APS	11 (37.9%)	7 (17.5%)	0.056
Second-degree relative with APS	4 (13.8%)	8 (20%)	0.502
Classification criteria 2006	27 (93.1%)	4 (10%)	<0.001
Classification criteria 2023	28 (96.5%)	1 (2.5%)	<0.001
Any cardiovascular risk factor	0 (0%)	0 (0%)	NA
Death	2 (6.9%)	0 (0%)	0.09

APS, antiphospholipid syndrome; NA, not available; NR, not reported; SLE, systemic lupus erythematosus.

**Table 2:** Clinical Manifestations - APS Vs Controls

Parameter	APS (n=29)	Control (n=40)	P value
Arterial thrombosis	10 (34.4%)	1 (2.5%)	<0.001
Venous thrombosis	20 (69%)	3 (7.5%)	<0.001
Unprovoked venous thrombosis	18 (62.1%)	0 (0%)	<0.001
Obstetric morbidity	2 (6.9%)	0 (0%)	0.09
Microvascular involvement	12 (41.4%)	4 (10%)	<0.001
Livedo reticularis	8 (27.9%)	0 (0%)	<0.001
Nephropathy	4 (13.8%)	5 (12.5%)	0.834
Catastrophic APS	3 (10.3%)	0 (0%)	0.035
Pulmonary haemorrhage	3 (10.3%)	0 (0%)	0.035
MINOCCA	0 (0%)	0 (0%)	NA
Adrenal haemorrhage	1 (3.4%)	0 (0%)	0.235
Heart valve disease	4 (13.8%)	0 (0%)	0.019
Thrombocytopenia (<130 000)	18 (62%)	14 (35%)	0.028
Platelets number	106±60	163±96.9	0.262

APS, antiphospholipid syndrome; NA, not available.

We evaluated the diagnostic utility of the 2006 and 2023 classification criteria using sensitivity, specificity and likelihood ratios (LR). The 2006 criteria showed a sensitivity of 87.1%, specificity of 94.7%, while the 2023 classification criteria demonstrated sensitivity of 96.6%, specificity of 97.5%. The 2023 ACR/EULAR criteria demonstrated a superior diagnostic profile, characterised by an LR+ of 18.23 compared with 16.43 for the 2006 Sydney criteria. This indicates that a positive result under the 2023 criteria is over 18 times more likely to occur in a patient with APS than in a control. Furthermore, the LR-decreased significantly from 0.136 to 0.036 with the 2023 criteria. This lower negative LR highlights the enhanced capacity of the new criteria to effectively rule out APS when the classification requirements are not met.

Interestingly, improved sensitivity and specificity of the 2023 classification criteria could be attributed to the fact that provoked thrombosis had a lower weight in the criteria compared with the 2006 version. Conversely, among patients who were considered to have APS, one patient did not fulfil the 2023 criteria, given high titre APL IgM subtype antibodies (which are not sufficient for the laboratory criteria) and thrombotic event. Finally, non-thrombotic manifestations were significantly more prevalent in the APS group; in our cohort, all patients with concomitant livedo reticularis (27.9% vs 0, p<0.001), heart valve disease (13.8% vs 0%, p=0.019) and diffuse alveolar haemorrhage (10.3% vs 0%, p=0.035) had APS syndrome; which may suggest that these non-thrombotic manifestations carry a significant diagnostic signal for APS within this paediatric cohort.

When evaluating laboratory findings (table 3), IgG isotype, both aB2GPI and aCL, were higher in the disease group (aB2GPI 79.3% vs 57.5%, p=0.033 and aCL 79.3% vs 50%, p=0.011). Interestingly, LA positivity did not differ significantly with our control group. Notwithstanding, the most specific serological marker for APS was triple APL positivity (ie, aCL and aB2GPI and LA)—with 69% of the APS group being triple positive (vs 40% in control group, p=0.001). aGAPSS was also significantly higher in our paediatric APS group (13±3.3 vs 9±4.1, =0.005). Lastly, antibodies toward red blood cells (direct agglutination—oombs) were more present in the APS group.

**Table 3:** Serology - APS Vs Controls

Parameter	APS (n=29)	Control (n=40)	P value
B2 GPI IGG	23 (79.3%)	23 (57.5%)	0.033
B2 GPI IGM	7 (24.1%)	11 (27.5%)	0.298
ACL IGG	23 (79.3%)	20 (50%)	0.011
ACL IGM	7 (24.1%)	9 (22.5%)	0.875
LAC	24 (82.8%)	31 (77.5%)	0.404
Single positive	6 (20.6%)	19 (47.5%)	0.043
Double positive	3 (10.3%)	7 (17.5%)	0.501
Triple positive	20 (69%)	14 (40%)	0.007
ANA	17 (58.6%)	27 (67.5%)	0.571
DSDNA	12 (41.4%)	14 (35%)	0.519
C3	92.5 (±22.2)	98 (±35.5)	0.853
C4	15.6 (±9)	18.8 (±10.3)	0.189
Coombs positive	13 (44.8%)	9 (22.5%)	0.038
aGAPSS	13 (±3.3)	9 (±4.1)	0.005

ACL, anti-cardiolipin; aGAPSS, adjusted Global Antiphospholipid Syndrome Score; LAC, lupus anticoagulant.

To evaluate the independent contribution of laboratory and clinical variables to the diagnosis of APS, a multivariate logistic regression model was constructed using the GAMLj framework (table 4). The model demonstrated high explanatory power (R^2^=0.56) and superior goodness-of-fit (AIC 52.79), effectively discriminating between APS cases and controls.

**Table 4:** Multivariate logistic regression analysis using globalised linear model

Predictor variable	OR	95% CI	P value
aGAPSS score	1.053	1.031 to 1.076	<0.001
Unrovoked VTE	1.554	1.025 to 2.356	0.038
Platelet count	0.999	0.998 to 1.000	0.035

Predictive accuracy: The model demonstrates a very high R2 of 0.557, which indicates that the combination of these laboratory and clinical parameters explains nearly 56% of the variance in APS outcomes.

aGAPSS, adjusted Global Antiphospholipid Syndrome Score; APS, antiphospholipid syndrome; VTE, venous thromboembolic.

The aGAPSS emerged as a highly significant independent predictor (OR 1.053; 95% CI 1.031 to 1.076; p<0.001), indicating that each unit increase in the score corresponds to a 5.3% increase in the odds of a positive diagnosis. Among clinical manifestations, unprovoked VTE remained a robust independent predictor, nearly doubling the diagnostic odds (OR 1.554; 95% CI 1.0205 to 2.356; p=0.007). Furthermore, a significant inverse association was observed between platelet count and APS status (p=0.033, OR 0.999), confirming that progressive decrements in platelet values independently contribute to an increased probability of the syndrome. While univariate analysis showed no significant difference in mean platelet counts between groups (p=0.226), platelet count emerged as a significant independent predictor in the multivariate model (p=0.033). This suggests that after adjusting for laboratory risk scores (aGAPSS) and thrombotic history, lower platelet values provide an independent and significant diagnostic signal for APS in this paediatric cohort

## Discussion

APS is a rare disease overall, and even more so in the paediatric population.^8^ Hence, diagnosis of this autoimmune disease in this population can be challenging as all classification criteria were originally developed for adults. Children offer a particularly intriguing context for understanding APS, as two of the most well-known aggravating factors—cardiovascular risk factors and pregnancy—are rarely present in this group. This may introduce bias in clinical practice and potentially lead physicians to misdiagnose APS in children.^13^

Several paediatric APS cohorts were previously published, of which the largest one was based on an international registry and included 121 patients.^14^ Others were small retrospective ones that focused mainly on presentation and clinical manifestations,^15^,^17^ thus necessitating more data on APS in children. Our study, although small and retrospective, is the first, to the best of our knowledge, to compare APS to aPL-positive-‘only’ patients (ie, per clinician assessment) in this population. Moreover, this is one of the first cohorts in which the new ACR-EULAR APS classification criteria were assessed in this age group.^18^

The results of our cohort provide several insights. First, APS among first-degree relatives is far more common among APS patients compared with aPL-controls (37.5% vs 17.5%, p=0.056). This finding shows only a trend and not true statistical significance, and thus should be interpreted with caution. Although APS is not typically associated with a familial inheritance pattern, the higher prevalence observed in this cohort suggests that clinicians should be vigilant in evaluating first-degree relatives of paediatric patients for the syndrome. If this trend is confirmed, a more proactive screening approach for family members may be warranted in paediatric cases. Genetic links in APS were delineated via whole genome sequencing, with loci found in HLA DRA, STAT1-STAT4, as well as interferon regulating genes,^19^ in the future researchers should consider genetic testing among paediatric. Second, arterial thrombosis is the APS defining event among paediatric population, interestingly enough. Following revision of all manifestations in a multivariate analysis arterial thrombosis and venous thrombosis are lone factors in determination of paediatric APS. Nevertheless, thrombocytopenia, microvascular involvement and heart valve disease were also more prominent and co-occurrence of all three was highly related to APS diagnosis in children. Serology associated with APS in this age group was mainly with IgG isotypes of aCL and aB2GPI as well as with triple positivity. Interestingly, LAC which is widely considered the most specific among aPLs, was similar among groups of children in our cohort.^20 21^

One of our main goals was to evaluate performance of the different classification criteria of APS in comparison to the clinical diagnosis of the treating specialists. Both the revised Sapporo criteria and the ACR-EULAR criteria perform well in children, with the ACR-EULAR criteria demonstrating superior performance, mainly due to higher sensitivity (96.1% vs 87.1%). Interestingly, in adult populations, the ACR-EULAR criteria perform better partly because they incorporate non-thrombotic manifestations^22^ while in this cohort, the higher sensitivity appears to result from a reduced emphasis of provoked thrombosis. In other words, in comparison to the 2006 criteria isolated provoked thrombosis in the presence of antiphospholipid antibodies may not necessarily lead to classification as APS.

The prevalence of catastrophic APS (CAPS) in the paediatric population is still unclear, as well as the prognosis of these patients. Of note, the rate of CAPS in this cohort was 10.3%. In adults, the prevalence of CAPS is much lower, the largest cohort published states that the prevalence is around 1%,^23^ while more recent publications of, granted smaller cohorts, state the prevalence tends to be a bit higher—around 3%,^24 25^ the rate of CAPS in previous paediatric cohorts is similar to ours and suggests that the rate of CAPS is higher among children.^26^ Moreover, previous reports in adults suggest that infections may serve as precipitating factors, with CAPS sometimes being the initial presentation.^26^ Conversely, in our study, all cases of CAPS were spontaneous, with no identifiable triggers noted, and only one patient presented with CAPS as the initial manifestation of APS.

Mortality was documented in 2 (6.9%) patients in the study period and both patients were within the APS group. One of which was diagnosed with CAPS and the other with ventilator-associated pneumonia following haemorrhagic transformation of ischaemic stroke. Mortality among paediatric patients in reported cohorts is considerable, one meta-analysis assessed the mortality rate at 7% (95% CI 4% to 11%).^27^ In contrast, reports of mortality among adults with APS are significantly lower, ranging from 1.8% to 2.8% in 10 years follow-up.^24 28^ The observed discrepancy in mortality rates between paediatric and adult patients highlights the urgent need to identify the subset of patients who are resistant to therapeutic interventions. Direct comparisons between paediatric and adult cohorts are often limited by heterogeneous follow-up and varying comorbidities. Furthermore, while the observed mortality rate in this specific cohort reflects a notable disease burden, these figures should be interpreted with caution due to the small sample size and require confirmation through larger longitudinal studies.

This study is not without limitations derived from its retrospective nature and relatively small size, typical for rare diseases in general and in children in particular. Another notable limitation is the composition of the control group. The inclusion of aPL-positive patients with underlying systemic diseases was necessary due to the rarity of asymptomatic paediatric carriers, yet this may limit the generalisability of our findings to the broader paediatric population. While this approach introduced potential confounding variables, it was deemed more robust than proceeding without a comparative cohort. Furthermore, the control group exhibited a high rate of triple positivity—a high-risk profile—suggesting that the development of APS cannot be entirely ruled out in these subjects. Consequently, further validation is required in broader paediatric populations, stratified into specific clinical subgroups.

Acknowledgeably, some aPL testing occurred in clinical scenarios with low pretest probability for thrombosis. While this potentially influences the criteria’s performance, it accurately reflects the frequent use of aPL screening in real-world paediatric autoimmune workups. Lastly, we cannot exclude selection bias as patients were diagnosed with APS by expert opinion in a ‘real-life setting’. Nonetheless, we believe that this well-selected cohort, followed for up to 15 years by a team of specialists, may enable important insights into paediatric APS.

## Conclusions

In this study, we assessed clinical and laboratory findings in a controlled cohort of paediatric APS. The diagnosis of APS was associated with similar diagnoses in a first-degree relative, thrombosis (both arterial and venous), microvascular involvement (eg, heart valve disease and thrombocytopenia). Triple positive APS antibody was the most prominent laboratory finding. Among our APS patients, mortality and CAPS rates were 6.9% and 10.3%, respectively. When assessing The ACR-EULAR in comparison to revised Sapporo, the former showed superior diagnostic, characterised by a higher positive LR (18.23 vs 16.43) and a lower negative LR (0.036 vs 0.136), effectively improving both the rule-in and rule-out capacity for APS diagnosis. Our data suggest that the 2023 ACR-EULAR criteria provide an effective framework for identifying paediatric APS, though their application should ideally be balanced with expert clinical judgement until larger, multicentre validations are conducted.

## Data Availability

Data are available on reasonable request.
